# Stable Isotopes Reveal Rapid Enamel Elongation (Amelogenesis) Rates for the Early Cretaceous Iguanodontian Dinosaur *Lanzhousaurus magnidens*

**DOI:** 10.1038/s41598-017-15653-6

**Published:** 2017-11-10

**Authors:** Celina A. Suarez, Hai-Lu You, Marina B. Suarez, Da-Qing Li, J. B. Trieschmann

**Affiliations:** 1 Department of Geosciences, University of Arkansas, 216 Gearhart Hall, Fayetteville, AR 72701 USA; 2 Key Laboratory of Vertebrate Evolution and Human Origins of Chinese Academy of Sciences, Institute of Vertebrate Paleontology and Paleoanthropology, Chinese Academy of Sciences, 142 Xizhimenwai Avenue, Beijing, 100044 P.R. China; 3 College of Earth Sciences, University of Chinese Academy of Sciences, Beijing, 100049 China; 4 Department of Geological Sciences, University of Texas at San Antonio, San Antonio, Texas 78249 USA; 5 Institute of Vertebrate Paleontology, Gansu Agricultural University,1 Yingmencun, Anning District, Lanzhou City, Gansu Province 730070 China

## Abstract

*Lanzhousaurus magnidens*, a large non-hadrosauriform iguanodontian dinosaur from the Lower Cretaceous Hekou Group of Gansu Province, China has the largest known herbivorous dinosaur teeth. Unlike its hadrosauriform relatives possessing tooth batteries of many small teeth, *Lanzhousaurus* utilized a small number (14) of very large teeth (~10 cm long) to create a large, continuous surface for mastication. Here we investigate the significance of *Lanzhousaurus* in the evolutionary history of iguanodontian-hadrosauriform transition by using a combination of stable isotope analysis and CT imagery. We infer that *Lanzhousaurus* had a rapid rate of tooth enamel elongation or amelogenesis at 0.24 mm/day with dental tissues common to other Iguanodontian dinosaurs. Among ornithopods, high rates of amelogenesis have been previously observed in hadrosaurids, where they have been associated with a sophisticated masticatory apparatus. These data suggest rapid amelogenesis evolved among non-hadrosauriform iguanodontians such as *Lanzhousaurus*, representing a crucial step that was exapted for the evolution of the hadrosaurian feeding mechanism.

## Introduction

**Figure 1:**
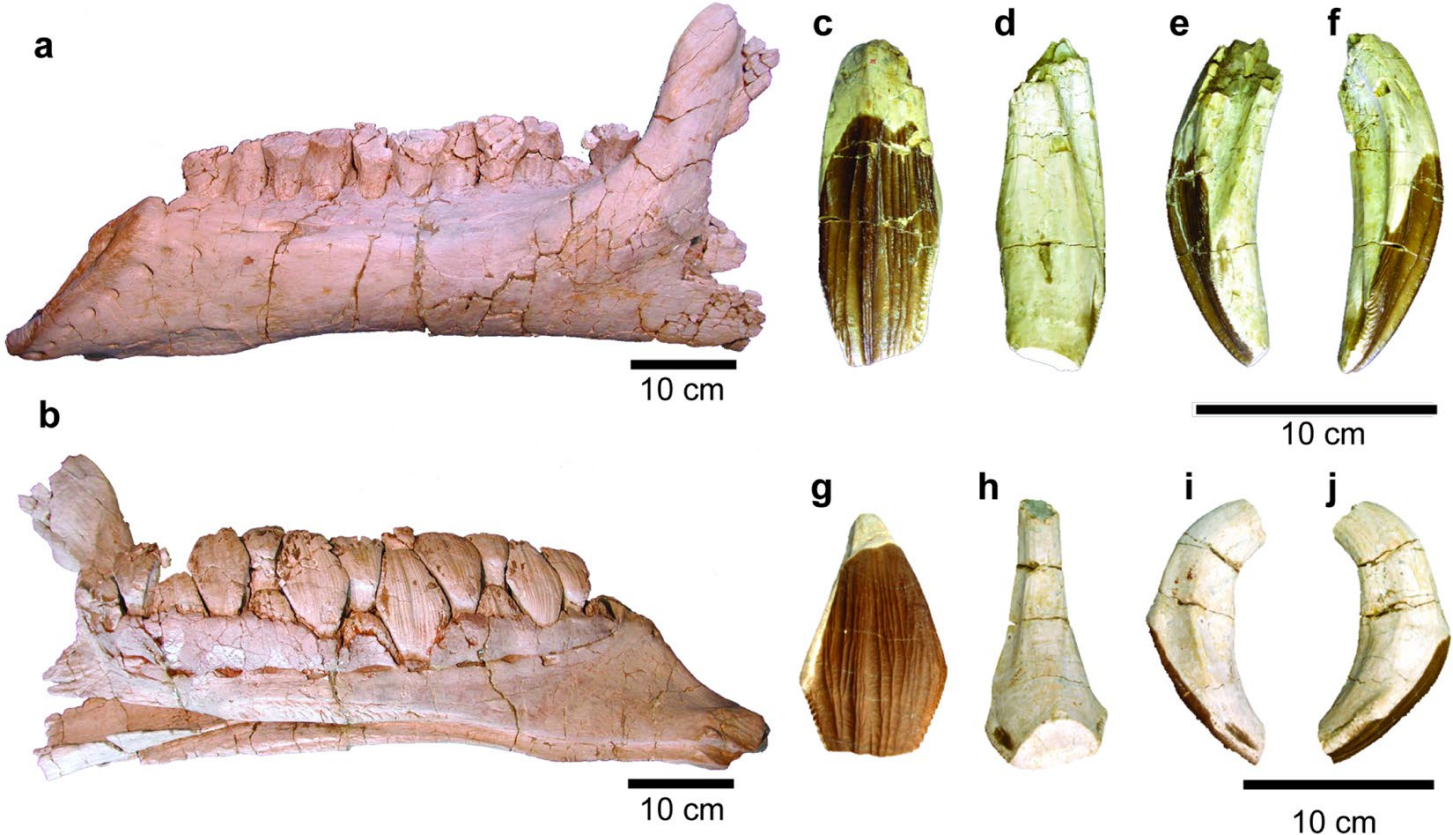
Jaw and Teeth of *Lanzhousaurus magnidens*. (**a**) Left dentary in lateral view and (**b**) medial view. Right maxillary tooth sampled in this study in (**c**) labial, (**d**) lingual, (**e**) mesial and (**f**) distal views. Right dentary tooth sampled in this study in (**g**) lingual, (**h**) labial, (**i**) distal, and (**j**) mesial views. Scale bar = 10 cm.

The geologic record of northern China provides an almost continuous record of terrestrial deposition from the Early Jurassic through the Late Cretaceous. This record documents changing fauna and climatic conditions in a series of intermontane basins. Global palaeontological evidence suggests that there was a rapid dinosaur faunal turnover during the first 30 Myr of the Cretaceous (Valanginian to the Aptian-Albian), when sauropod- and basal iguanodontian-dominated ecosystems were replaced by neoceratopsian- and hadrosauroid-dominated ecosystems^1,2^. These changes, along with the evolution and widespread diversification of angiosperms, pollinating insects, mammals and birds, are known as the Cretaceous Terrestrial Revolution (KTR)^2–5^ and may correlate with major climatic shifts in the Early to mid-Cretaceous. One of the enigmatic dinosaurs preserved during this time is the derived non-hadrosauriform iguanodontian *Lanzhousaurus magnidens* ^6^. This animal preserves the largest ornithischian dinosaur teeth recorded at over 10 cm long (Fig. 1). Whereas more derived hadrosauroids evolved a greater number of relatively small teeth to form a dental battery, this animal only maintained a small number (14 alveoli per jaw quadrant) of very large teeth, housed in meter-long jaws. This dichotomy in tooth number and size difference for hadrosauroids and *Lanzhousaurus* provokes several biologic and environmental questions: First, what if any seasonal records can be detected in these teeth? Such large teeth offer the potential for long, multi-year records. Second, if a seasonal record is preserved, we can calculate enamel elongation (amelogenesis). Did these animals lay down enamel rapidly or slowly? Here, we use stable isotopic composition of serially sampled teeth of the holotype of *Lanzhousaurus* to answer these questions. The ultimate test of tooth growth rate would be to thin section the teeth and count lines of von Ebner. However, given the rarity of these teeth, thin sectioning is not possible, precluding the ability to count von Ebner lines. Thus, less damage is incurred on these unique teeth by using stable isotopic analysis, allowing us to infer tooth elongation (rather than growth rate). Below, we use stable isotope data to infer rapid amelogenesis for *Lanzhousaurus*. We test these inferred amelogenesis rates using CT imaging, which revealed long-period or Andresen lines^7,8^ whose thickness is consistent with the rapid rates of amelogenesis derived from stable isotope patterns. CT imaging also revealed dentine tissue types consistent with the phylogenetic placement of *Lanzhousaurus* such as orthodentine and secondary dentine. We contextualize our findings phylogenetically to show the importance of rapid tooth elongation in the evolution of Styracosterna as it diversified to become one of the predominate herbivorous clades of the Late Cretaceous^9,10^.

## Regional Setting

The Hekou Group was deposited near Lanzhou City, north-central China, in an intracontinental rift basin that resulted from the Qilian Mountain polycyclic orogenic belt, and represents almost the entire Early Cretaceous from Berriasian to Albian^11,12^. Sediment was sourced from the southern Qilian Mountains. At its base, the Hekou Group is composed of thick alluvial conglomerates; it fines upward to interbedded fluvial-deltaic sandstones and calcic red-bed paleosols, and is capped with lacustrine sands and shales at the top of the group. *Lanzhousaurus magnidens* was discovered above the basal thick conglomerates at the base of a channel sandstone that cuts through a series of calcic paleosols. Recent carbon-isotope chemostratigraphic work by Suarez *et al*.^13^ suggest that the Hekou Group where *Lanzhousaurus* was recovered is late Barremian to Aptian in age. *Lanzhousaurus* teeth were sampled for phosphate oxygen isotopes to assess seasonality and enamel amelogenesis. In an attempt to resolve the relationship between amelogenesis rate and tooth growth rate we also conducted preliminary CT imaging of the sampled teeth.

## Results

**Figure 2:**
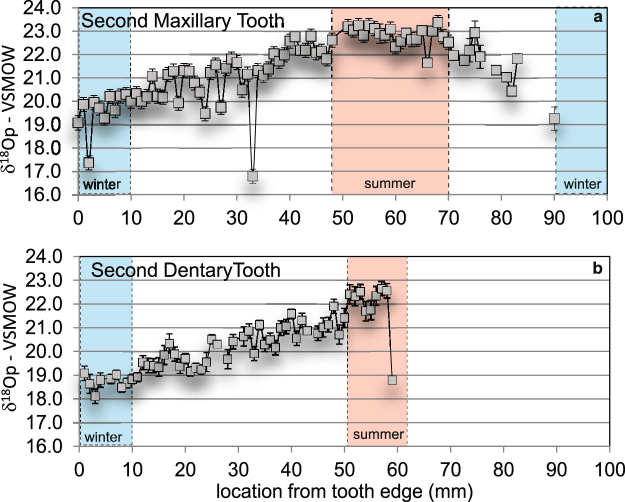
Results of isotopic analysis for tooth and bone phosphate and carbonate. (**a**) Maxillary  tooth and (**b**) dentary  tooth record with respect to location along the tooth from the tip (0 mm) to the base of the tooth. The sample at 0 mm represents the oldest portion of the tooth. Blue bars represent low O-isotopic composition consistent with winter values in a typical non-monsoonal temperate climate and light red bars represent enriched ^18^O values typical of summer season isotopic values. Error bars represent 1σ.

Results of oxygen isotope values (Table 1) are reported relative to Vienna Standard Mean Ocean Water (VSMOW). Phosphate oxygen isotopic composition (δ^18^O_p_) of *Lanzhousaurus* has a cyclic pattern when plotted relative to sample location along the long axis of the tooth. The average δ^18^O_p_ of all *Lanzhousaurus* tooth samples (both maxillary and dentary) averages 20.94 ± 1.42 1σ (Table 1, Supplemental material). Average δ^18^O_p_ for the maxillary tooth relative to the dentary tooth are not greatly different from each other (21.37 ± 1.39‰ *vs*. 20.30‰ ± 1.21‰); however they are significantly different from each other at α = 0.05; p ≪ 0.05 (two-tailed t-test). This difference may be the result of comparing incomplete tooth records. When comparing the first half of the record to each other (0 to 59 mm), the teeth are still significantly different from each other but with a greater p value (p = 0.0001). The maximum variability (discounting likely outliers) for both teeth is similar: 4.53‰ for the dentary and 4.30‰ for the maxillary tooth. Enamel δ^18^O_p_ for both the dentary and the maxillary tooth show a gradual increasing trend across the first ~50 mm as measured from the tip. For the maxillary tooth, δ^18^O_p_ reaches a maximum value of 23.37‰ at 68 mm and then decreases to the end of the tooth record (Fig. 2). This suggests a ~4.5‰ seasonal trend recorded in the teeth. Assuming that the long-term trend in the δ^18^O_p_ of *Lanzhousaurus* represents a single one year cycle and the total tooth enamel length is ~90 mm, then the tooth enamel elongation rate (amelogenesis rate) of *Lanzhousaurus* was 90 mm/ yr. (0.24 mm/day) or 240 μm/day (accounting for 371 days in a Cretaceous year^14^).

**Table 1:** Summary of results for isotopic analysis of oxygen and carbon. All oxygen isotopic compositions are relative to V-SMOW.

sample	n = (sample size)	avg. δ^18^O_p_	1σ δ^18^O_p_
*L*. *magnidens* dentary tooth	55	20.3	1.2
*L*. *magnidens* maxillary tooth	79	21.4	1.4

**Figure 3:**
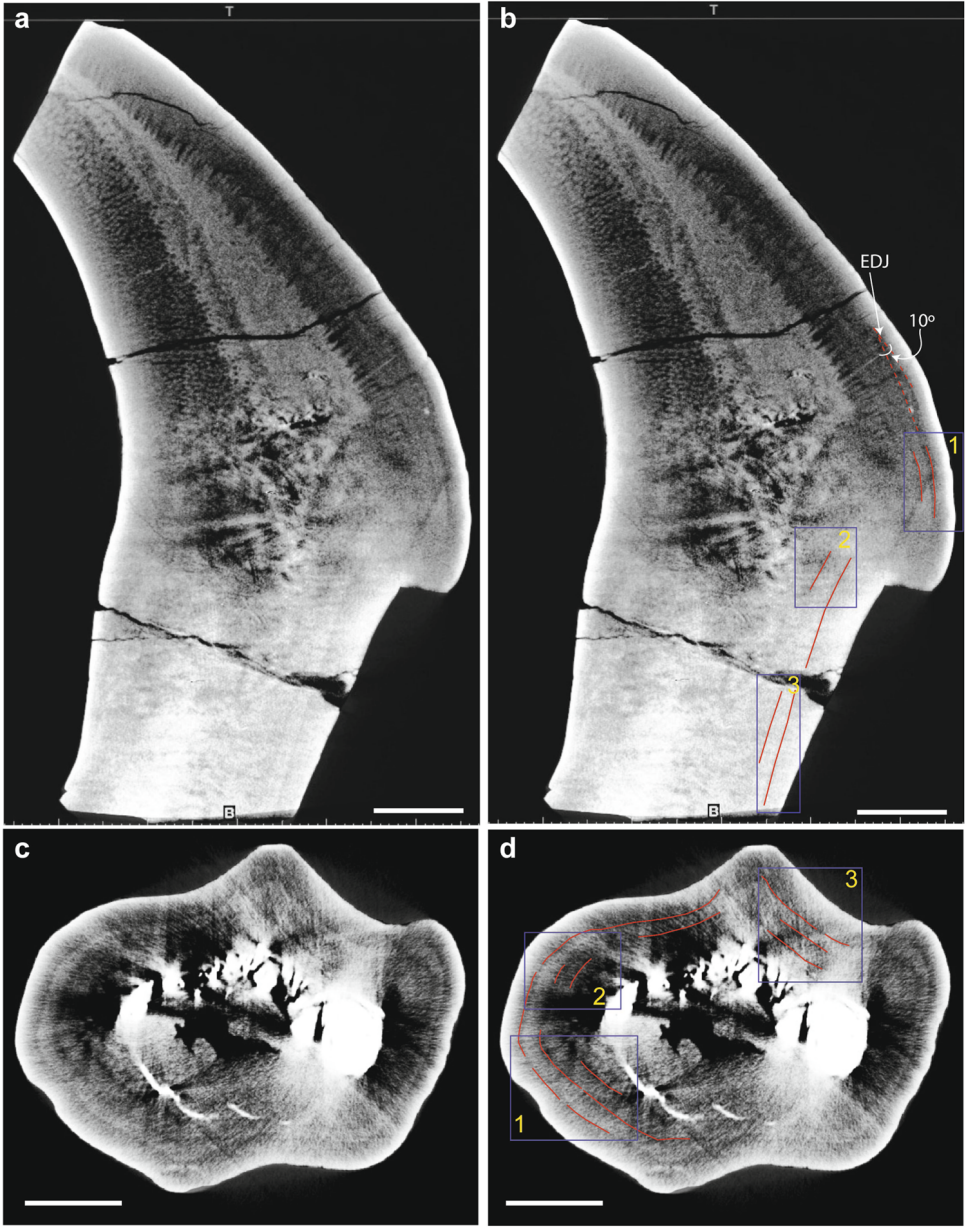
CT images of dentary tooth in (**a**) coronal view, (**b**) coronal view with incremental lines measured in three regions. Measured angle of the incremental line relative to the enamel-dentine juncture (EDJ) is ~10° (**c**) axial view and (**d**) axial view with incremental lines measured in three regions. Scale bar = 1 cm.

Micro CT images using 225 kV micro-computerized tomography (225 micro-CT) (developed by the Institute of High Energy Physics, Chinese Academy of Sciences) at the Key Laboratory of Vertebrate Evolution and Human Origins, Chinese Academy of Sciences, reveal at least three tooth tissues within the dentine (Fig. 3). Unfortunately, the resolution of the scans is insufficient for recognition of daily deposited von Ebner lines; however, several long-period incremental lines were observed. The three tissues observed include (1) a dense (light colored) core, (2) a porous and less dense (dark) tissue on the side opposite to the enamel coating, and (3) a tissue with horizontal striations relative to the transverse plane and perpendicular to the long tooth axis, that mantles the enamel side of the tooth. It is in this tissue that long period incremental lines are observed. Where the lines were well-preserved, they were measured using scaled Adobe Illustrator ® files and the measurement tool. A total of 130 measurements between both teeth (see supplemental data table) resulted in an average thickness of the incremental lines of 1.736 ± 0.311 mm (1σ).

## Discussion

The isotopic composition of tooth phosphate is the more robust of the two main components of teeth; phosphate and carbonate^15^. The O-isotopic composition of both molecules can be compared to determine if diagenetic overprinting has taken place. If δ^18^O_CO3_ values are the same as δ^18^O_p_ values it may suggest overprinting. This is not the case (see electronic supplemental material). Deviation from theoretical equilibrium would also suggest alteration to one or both O-isotope values^16^. Relative to bone, tooth enamel crystallites are much larger and contain much less organic matter, restricting alteration to the phosphate molecule^17^. Thus, tooth δ^18^O_p_ values likely represent primary biogenic values. Additionally, if diagenesis had reset δ^18^O_p_, there would be no trend in the data, rather it would converge on a single value or have a wide scatter with no trend at all^18–21^. Finally, the sinusoidal trend could only represent seasonality or seasonal migration, since an ontogenetic trend such as a change in metabolism^22,23^ would result in a directional trend rather than a cyclic trend.

Given the authenticity of the isotope values, the sinusoidal trend seen in the isotope time series taken from *Lanzhousaurus* is reminiscent of similar seasonal or migratory trends in the isotopic composition of ingested water observed in mammal and dinosaur time-series analyses over the course of up to a full year^20,24–26^. Given this interpretation, the δ^18^O_p_ values from *Lanzhousaurus* preserve isotopic signals associated with seasonal or migratory fluctuations as recorded during the yearly formation of enamel. The long-term trend from low isotopic composition to higher isotopic composition and then back to lower isotopic composition (Fig. 2) of the tooth suggests a winter to summer to winter cycle observed in other dinosaur teeth and mammals^20,24^. Three samples are anomalously low, (maxillary tooth at 3 mm, 33 mm, and dentary tooth at 59 mm), representing ~2% of the data. These points are considered outliers and an artifact of instrumentation error (see supplementary material). Given these points, if the final sample at 90 mm on the maxillary tooth also represents a statistical outlier, it would call into question the winter signal in the youngest portion of the tooth. However, even with the removal of the final sample at 90 mm, there is a clear decreasing trend in the isotopic composition of tooth enamel for the youngest part (~70–90 mm) of the second maxillary tooth. If this point is removed, the cyclic trend still is evident and the tooth represents a little less than a full year.

Other authors have used offsets as low as 1.73‰ to as high as 9‰ to suggest seasonal variations^24–27^. Our data represent moderately seasonal variability for a C_3_-dominated ecosystem. Straight *et al*.^25^ documented seasonal trends on the basis of a 1.93‰ maximum variability of δ^18^O_p_ and suggested that the low variability represented humid, temperate conditions during the deposition of the Horseshoe Canyon Formation, Alberta, Canada. Koch^26^, on the other hand, reported high seasonal variability in mastodon and mammoth tusks of 9‰, suggesting that such seasonal variation was controlled by extreme temperature and humidity changes during glacial periods. *Lanzhousaurus* either seasonally migrated to a different location with different water isotopic composition, or teeth recorded locale-specific seasonal changes in precipitation δ^18^O. Without analysis of other animals or migration proxies such as Sr-isotope analysis, it is impossible to determine the cause of the seasonal trend. Nonetheless, the δ^18^O_p_ values from *Lanzhousaurus* suggest that it experienced seasonal changes common in modern semi-arid to semi-humid environments. This is consistent with sedimentologic evidence (cummulic red-bed paleosols, abundant pedogenic carbonate nodules) collected from the formation.

This seasonal trend allows us to calculate the tooth enamel elongation rate (rate of amelogenesis) of *Lanzhousaurus*. It is important to note that we are not calculating tooth growth rate, replacement rate, or wear rates using this method, but rather the rate at which enamel was laid down relative to the long axis of the tooth. Erickson^28^ showed that daily growth lines contained within tooth dentine suggest that dinosaur tooth growth rates (and likely vertebrates in general) did not exceed ~30 μm/day or 10.95 mm/yr. Rates calculated on the basis of stable isotopes are not tooth growth rates but rather rates of enamel mineralization or amelogenesis relative to the long axis of the tooth. Enamel elongation rates differ from dentine apposition rates measured by incremental lines of von Ebner because ameloblasts and odontoblasts lay down material at different angles relative to the long axis of the tooth. The shallower the angle that mineral layers are laid down relative to the tooth axis, the greater the rate of elongation^29^. For example, animals with rapidly elongating teeth like beavers and elephants (tusk) only lay down 30 μm of dentine per day, yet can lay down several millimeters to 100 s of centimeters of dentine (respectively) along the axis of the tooth per day. Previous isotopic studies have referred to enamel elongation rate as “tooth formation rate,” but the latter term can also refer to the rate at which dentine or enamel is lain down parallel to fronts of apposition. To avoid this ambiguity, we recommend avoiding the term “tooth formation rate.” For example, Straight *et al*.^25^ and Thomas and Carlson^20^ used similar isotopic method to determine the “growth rate of teeth.” These studies actually calculated enamel elongation. Straight *et al*.^25^ document similar “enamel elongation rates” of tyrannosaurid teeth as has been calculated on the basis of von Ebner line thickness by Erickson^28^. Thomas and Carlson^20^ suggested that *Edmontosaurus* “enamel elongation rate” was approximately 38 mm/ yr. or 0.1 mm/day or 100 μm/day, triple the maximum of dentine formation rates for all dinosaurs and amniotes in general calculated by Erickson^28^ (30 μm/day) on the basis of von Ebner lines. In the case of Thomas and Carlson^20^, the rate calculated suggests that enamel is laid town at a very shallow angle relative to the enamel-dentine juncture. The *Lanzhousaurus* specimens represent a single one year cycle, suggesting a tooth enamel elongation rate (amelogenesis rate) of 90 mm/yr. (0.24 mm/day) for *Lanzhousaurus*. This is nearly 2.5 times faster than rates calculated by Thomas and Carlson^20^ for *Edmontosaurus*. Thus, *Lanzhousaurus magnidens* has one of the fastest recorded tooth enamel elongation rates recorded for any iguanodontian dinosaur. This rapid rate suggests the angle of mineralization relative to the tooth axis is very shallow. CT images of growth bands support this calculation (see below).

**Figure 4:**
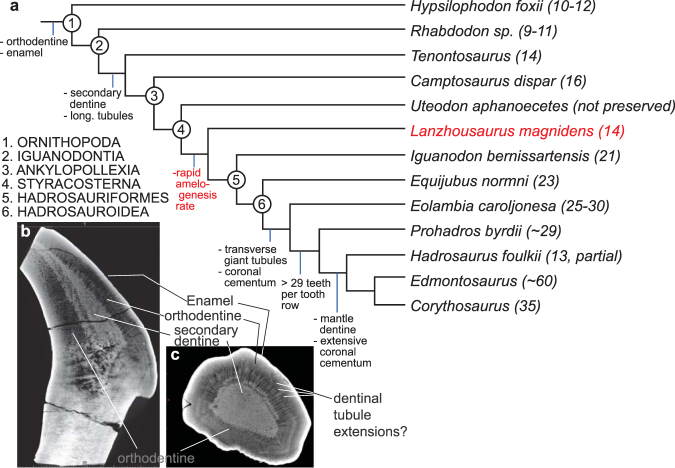
Evolution of dental characteristics from Erickson *et al*.^10^ and this study superimposed on (**a**) the phylogeny of McDonald *et al*.^31^. The numbers after the taxon names represent the number of tooth alveoli per too row. Tooth tissues described in Erickson *et al*.^10^ are identified in the transverse CT section (**b**) and axial (**c**) of the *Lanzhousaurus* dentary tooth.

Polarized microscopy of tooth thin sections is necessary to assess the histology of these teeth before definitive identification of the dental tissues can take place. Despite this, and the less than optimal resolution of CT images, we observe several tissue types as well as incremental lines within the teeth (Fig. 3 and additional images in supplemental data file) and give the following preliminary identifications: (1) The porous tissue on the opposite side of the tooth relative to the enamel is interpreted as orthodentine with dentinal tubules angled obliquely to the plane of the image. (2) The outer tissue on the enamel side of the tooth is interpreted as orthodentine and the transverse structures are thought to be pulp cavity with dentinal tubule extensions where orthodentine has not completely mineralized. Several growth lines are observed within the orthodentine at a very low angle (5 to 10°) to the enamel-dentine juncture (Fig. 4, Supplemental Fig. S2). (3) The core of dense mineral is interpreted as either poorly mineralized orthodentine (due to the lighter hue of the tissue in CT images) or could be secondary dentine that has filled the pulp cavity^10^. Given that in most ornithopod teeth, secondary dentine is bounded by orthodentine, it is likely that the inner core is secondary dentine that has filled the pulp cavity^10^ and *Lanzhousaurus* teeth contain typical dental histology of other members of Iguanontia (Fig. 4).

Several regions within the orthodentine on both teeth (Fig. 3) where the lines were well-preserved were measured, for a total of 130 measurements between both teeth (see supplemental data table). The average thickness of the incremental lines was 1.736 ± 0.311 mm. These lines are far too thick to represent von Ebner lines. However, approximately weekly or long period incremental lines called Andresen lines have been observed in other archosaurs; Andresen lines typically have 7–13 von Ebner lines between them^7,8^ and a thickness as much as 275 μm. The incremental lines in *Lanzhousaurus* are six times thicker. These lines are much thicker than weekly growth lines in other archosaurs, and we therefore conservatively regard them as representing longer time intervals of unknown periodicity or cause. Nonetheless, the shallow angle at which these lines intersect the enamel-dentine juncture suggests rapid enamel amelogenesis (mineralization) rates relative to the tooth axis.

Rapid tooth growth and amelogenesis rates are known from duck-billed dinosaurs such as *Edmontosaurus* ^20^; however, in these more derived hadrosauroids rapid amelogenesis co-occurs with rapid growth (and replacement) rate of many small, narrow-crowned teeth. Tooth counts greater than 29 per tooth row, multiple tooth rows, and a total of up to 700 teeth (including several generations of unerupted teeth) are present in derived members of Hadrosauroidea. For example, You *et al*.^30^ showed that the derived character of greater than 29 alveoli per tooth row as well as a reduction in tooth size make its first occurrence ~96 million years ago, with such characters observed in *Protohadros byrdii*. The combination of rapid enamel elongation rate, reduced tooth size, and increased number of teeth eventually lead to specialization of tooth batteries used for either slicing, grinding, or both^10^. In addition, these smaller teeth evolve more complex tooth tissues over time. Erickson *et al*.^10^ shows the progression of tooth tissue characters in hadrosaurid dinosaurs. Outgroup basal genasaurians have simple non-occluding teeth composed of enamel on both sides and orthodentine. Members of Ornithopoda such as *Tenontosaurus* and *Iguanodon* develop abscess preventing secondary dentine and longitudinal giant tubules with enamel on one side. This allowed their teeth to be self-sharpening. Members of Hadrosauroidea develop transverse giant tubules and coronal cementum. Finally, members of Hadrosauridae evolve wear-resistant mantel dentine and extensive coronal cementum allowing the formation of grinding surfaces with crests and ridges.

*Lanzhousaurus* has tooth tissue characters typical of Ornithopoda (Fig. 4) including enamel on one side, orthodentine, and secondary dentine. This is consistent with its phylogenetic placement^6,31^. In this case the added character of rapid amelogenesis is a unique by-product of allometry for these non-hadrosauriform iguanodontians (Fig. 4). *Lanzhousaurus*, as opposed to Hadrosauriformes, implemented rapid enamel mineralization rates for a small number of very large teeth (14 tooth alveoli per tooth row). Although quantitative replacement rates cannot be determined without direct evidence of von Ebner lines, we suggest the working hypothesis that replacement was slow since only one replacement tooth is observed within the jaw (Fig. 1).

This study helps clarify the progression of dental specializations among Ornithopoda and Hadrosauridae, while at the same time raising new questions. Figure 4 shows a modified iguanodontian-hadrosaurid phylogeny by McDonald^31^ with characters added from Erickson^10^ and You^30^. *Lanzhousaurus* is the oldest iguanodontian dinosaur with documented rapid amelogenesis rates indicating that this evolutionary adaptation existed as early as in non-hadrosauriform iguanodontians. Future work to identify rates of tooth enamel amelogenesis within sister taxa to *Lanzhousaurus*, common ancestors to *Lanzhousaurus* and hadrosaurs, and advanced hadrosaurs are necessary to determine the exact timing of rapid tooth amelogenesis and if it is important in the evolution of hadrosaurs.

*Lanzhousaurus* evolved a method of rapid enamel elongation, large tooth size, and a small number of teeth that was ultimately unsuccessful relative to the advent of rapid growth of dental batteries as is seen in advanced hadrosaurids. Additional investigation of tooth enamel elongation rates is warranted to determine if it contributed to the character evolution leading to hadrosaurid dental specialization. Rapid tooth elongation rates, along with later derived adaptations to tooth tissues, tooth size (reduced), and tooth configuration (packed) allowed hadrosaurs to evolve specialized mastication techniques. These specializations allowed them to become the predominant large herbivores of the Late Cretaceous.

## Material and Methods

*Lanzhousaurus* teeth are from the holotype of *Lanzhousaurus magnidens* ^6^ and were found approximately 30 km south of Lanzhou City in the Lanzhou-Minhe Basin. The sampled teeth include a maxillary tooth (GSIVP00001-1) and one dentary tooth (GSIVP00001-2) from the holotype (Fig. 1 and Supplemental Figure S1). These samples are currently housed at the Institute of Vertebrate Paleontology, Gansu Agricultural University.

Detailed description of sampling and isotope analyses performed on all samples can be found in Supplemental electronic material. Briefly, *Lanzhousaurus* tooth enamel phosphate showed coloration bands that were spaced every one millimetre. We interpreted this to represent perikymata lines and so the tooth was sampled every one millimetre. Care was taken to only sample enamel and not dentine. Approximate 300 to 500 µg of enamel was sampled. The enamel phosphate was analysed using the silver phosphate method presented by O’Neil *et al*.^32^ and modified by Bassett *et al*.^33^ and analysed at the Keck Paleonenvironmental and Environmental Stable Isotope Laboratory at the University of Kansas.

X-ray CT scanning of the teeth was carried out by using 225 kV micro-computerized tomography (225 micro-CT) (developed by the Institute of High Energy Physics, Chinese Academy of Sciences) at the Key Laboratory of Vertebrate Evolution and Human Origins, Chinese Academy of Sciences (CAS). The specimens were scanned with beam energy of 150 kV and a flux of 130 μA at a detector resolution of 62.73 um per pixel using a 360° rotation with a step size of 0.5° and an unfiltered aluminum reflection target. A total of 720 transmission images were reconstructed in a 2,048*2,048 matrix using a two-dimensional reconstruction software developed by the Institute of High Energy Physics, CAS. The three-dimensional images of the teeth were assembled using the volume analysis software VGstudio Max 2.1 (Volume Graphics, Germany). Digitally shearing and editing of the specific images were conducted using Mimics 16.0 (Materialise, Belgium).

All data are available in the online supplemental data files.

## Electronic supplementary material

Supplementary Information and Data
