# Supplementary material for: Stable Isotopes Reveal Rapid Enamel Elongation (Amelogenesis) Rates for the Early Cretaceous Iguanodontian Dinosaur Lanzhousaurus magnidens
Source: Sci Rep. 2017 Nov 10;7:15319. doi: 10.1038/s41598-017-15653-6 (PMC5681512; doi:10.1038/s41598-017-15653-6)
Supplement: Supplementary file 1 — Supplementary Information and Data [file 41598_2017_15653_MOESM1_ESM.pdf]

Stable Isotopes Reveal Rapid Enamel Elongation (Amelogenesis) Rates for the Early Cretaceous Iguanodontian Dinosaur *Lanzhousaurus magnidens*

Authors: Celina A. Suarez, Hailu You, Marina B. Suarez, Da-Qing Li and J.B. Trieschmann

Supplemental Electronic Material

Supplemental Description of Teeth

The teeth sampled were found at the site of the holotype discovery of *Lanzhousaurus magnidens*. The two teeth sampled were isolated teeth from the quarry site. The dentary jaw discovered includes 14 teeth in the sockets. No maxilla was discovered at the site. Considering only one individual was discovered at the site, it likely that the two teeth sampled were from the same individual. Tooth dimensions can be found in Supplemental Data Table section below (Table S1).

Supplemental Detailed methods:

Phosphate analysis

Phosphate samples were drilled and analysed at University of Kansas Keck Paleoenvironmental and Environmental Stable Isotopes Laboratory (KPESIL). *Lanzhousaurus* tooth enamel is rather thick at about 0.5-0.75 mm. With such thick enamel we were able to control when the microdrill was approaching the dentine. The tooth enamel is orange in color so as the drill started to approach the enamel-dentine interface, the color started to get very light. When this occurred we stopped drilling to avoid sampling dentine. Samples were analysed on a High Temperature Conversion Elemental Analyzer (TC/EA) connected to a ThermoFinnigan MAT 253 continuous-flow IRMS at 1400°C. NIST-120c was used as a quality control for these samples, with an average $\delta^{18}O = 22.4 \pm 0.6‰$ V-SMOW. Instrument stability and precision was also monitored via the analysis of IAEA-601 benzoic acid and returned a values of 22.1 ± 0.4‰ V-SMOW. Turtle and crocodile $\delta^{18}O_p$ samples were analysed at the University of Arkansas Stable Isotope Laboratory (UASIL). Instrument stability and precision at UASIL was monitored via analysis of USGS-34, IAEA-C3 sucrose, and USGS-35 which returned values of -27.9 ± 0.5‰ V-SMOW, 57.5 ± 1.2‰ V-SMOW, and 37.2 ± 0.1‰ V-SMOW respectively. NIST 120c was used as a quality control standard and returned a value of 23.7 ±0.4 VSMOW.

Supplemental Data tables

Table S1: Tooth Dimensions

Tooth	Basal-Apical Length (mm)	Mesial-Distal Length (mm)	Lingual-Labial Length (mm)
Maxillary (GSIVP00001-1)	125	45	35
Dentary Tooth (GSIVP00001-2)	124	57	45

Table S2: All isotopic measurements

sample	Element	Location (mm)	$\delta^{18}O_p$-VSMOW	$\delta^{18}O_{CO3}$-VSMOW	$\delta^{13}C$-VPDB
LZSM-0	L. magnidens maxillary tooth	0	19.1		
LZSM-1		1	19.9		
LZSM-2		2	17.4		
LZSM-3		3	19.9		
LZSM-4		4	19.7		
LZSM-5		5	19.3		
LZSM-6		6	20.2		
LZSM-7		7	19.6		
LZSM-8		8	20.2		
LZSM-9		9	20.3		
LZSM-10		10	20.0		
LZSM-11		11	20.3		
LZSM-12		12	20.0		
LZSM-13		13	20.2		
LZSM-14		14	21.1		
LZSM-15		15	20.2		
LZSM-16		16	20.2		
LZSM-17		17	21.1		
LZSM-18		18	21.3		
LZSM-19		19	19.9		
LZSM-20		20	21.3		
LZSM-21		21	21.3		
LZSM-22		22	20.8	25.1	-7.6
LZSM-23		23	20.4		
LZSM-24		24	19.5		
LZSM-25		25	21.2	24.6	-6.0
LZSM-26		26	21.6		
LZSM-27		27	19.8	24.4	-6.3
LZSM-28		28	21.4		
LZSM-29		29	21.8	23.8	-6.0
LZSM-30		30	21.6		
LZSM-31		31	20.5	24.4	-5.9
LZSM-32		32	21.2		
LZSM-33		33	16.8	24.4	-6.5
LZSM-34		34	21.3		
LZSM-35		35	21.1	23.6	-6.2

sample	Element	Location (mm)	$\delta^{18}O_p$-VSMOW	$\delta^{18}O_{CO3}$-VSMOW	$\delta^{13}C$-VPDB
LZSM-36		36	21.4		
LZSM-37		37	22.0		
LZSM-38		38	21.7		
LZSM-39		39	22.0	24.2	-6.4
LZSM-40		40	22.6		
LZSM-41		41	22.8	23.6	-6.2
LZSM-42		42	22.2		
LZSM-43		43	22.2		
LZSM-44		44	22.8	24.5	-6.3
LZSM-45		45	22.1		
LZSM-46		46	22.1		
LZSM-47		47	21.8	23.4	-6.5
LZSM-48		48	22.6		
LZSM-51		51	23.2		
LZSM-52		52	23.0	23.7	-5.9
LZSM-53		53	23.2		
LZSM-54		54	22.8		
LZSM-55		55	23.2	24.8	-5.8
LZSM-56		56	23.1		
LZSM-57		57	22.9	25.8	-6.2
LZSM-58		58	22.8		
LZSM-59		59	23.1	25.2	-6.4
LZSM-60		60	22.3		
LZSM-61		61	22.6		
LZSM-62		62	22.9	25.5	-6.3
LZSM-63		63	22.7		
LZSM-64		64	22.7	26.4	-6.2
LZSM-65		65	23.0		
LZSM-66		66	21.7	25.1	-4.9
LZSM-67		67	23.0		
LZSM-68		68	23.4	25.5	-7.0
LZSM-69		69	22.8		
LZSM-70		70	22.5	21.8	-10.0
LZSM-71		71	22.0		
LZSM-73		73	21.8		
LZSM-74		74	22.2		
LZSM-75		75	22.9		
LZSM-76		76	21.9		
LZSM-79		79	21.3		
LZSM-81		81	21.0		

sample	Element	Location (mm)	$\delta^{18}O_p$-VSMOW	$\delta^{18}O_{CO3}$-VSMOW	$\delta^{13}C$-VPDB
LZSM-82		82	20.4		
LZSM-83		83	21.8		
LZSM-90		90	19.3		
Max variability			4.30	4.64	2.65
LZSD-1	L. magnidens dentary tooth	1	19.1		
LZSD-2		2	18.6		
LZSD-3		3	18.1		
LZSD-4		4	18.8		
LZSD-6		6	18.8		
LZSD-7		7	19.0		
LZSD-8		8	18.5		
LZSD-9		9	18.7		
LZSD-10		10	18.8		
LZSD-11		11	18.9		
LZSD-12		12	19.5	22.6	-5.4
LZSD-13		13	19.4		
LZSD-14		14	19.4		
LZSD-15		15	19.4	24.3	-6.1
LZSD-16		16	19.9		
LZSD-17		17	20.3		
LZSD-18		18	19.9	24.2	-6.0
LZSD-19		19	19.4		
LZSD-20		20	19.7		
LZSD-21		21	19.2	22.8	-6.1
LZSD-22		22	19.3		
LZSD-23		23	19.2		
LZSD-24		24	19.6	23.2	-6.4
LZSD-25		25	20.5		
LZSD-26		26	20.3		
LZSD-28		28	19.7	23.8	-6.6
LZSD-29		29	20.4		
LZSD-30		30		25.5	-5.8
LZSD-31		31	20.6		
LZSD-32		32	20.9		
LZSD-33		33	19.9	25.0	-6.1
LZSD-34		34	21.1		
LZSD-35		35	20.3		
LZSD-36		36	20.5		

sample	Element	Location (mm)	$\delta^{18}O_p$-VSMOW	$\delta^{18}O_{CO3}$-VSMOW	$\delta^{13}C$-VPDB
LZSD-37		37	20.2		
LZSD-38		38	21.0		
LZSD-39		39	21.1		
LZSD-40		40	21.6	26.0	-5.9
LZSD-41		41	20.6		
LZSD-42		42	21.3		
LZSD-43		43	20.9		
LZSD-45		45	20.8	25.9	-5.6
LZSD-46		46	21.0		
LZSD-47		47	21.1		
LZSD-48		48	21.9		
LZSD-49		49	20.7		
LZSD-50		50	21.4	24.4	-5.7
LZSD-51		51	22.4		
LZSD-52		52	22.3	24.9	-6.6
LZDS-53		53	22.5		
LZSD-54		54	21.7	24.5	-5.8
LZSD-55		55	21.7		
LZDS-56		56	22.3		
LZDS-57		57	22.6		
LZSD-58		58	22.6		
LZSD-59		59	18.8		
LZSD-60		60		24.5	-5.8
LZSD-64		64		24.9	-6.3
Max variability			4.53	3.4	1.2

Table S3: Statistical comparison of data

Comparison	F-test (Variance)	F-test P value	T-test	T-test P value
L. magnidens maxilla vs dentary tooth $\delta^{18}O_p$	Unequal variance	P = 0.0242	Significantly different	P << 0.05
L. magnidens maxilla vs dentary tooth $\delta^{18}O_p$ (first 59 mm only)	Equal variance	P = 0.054	significantly different	P = 0.0003

Table S4: Incremental Line Measurements (see figure S5)

Maxilla (coronal section)	Line 1 to 2 (mm)	Lines 2 to 3 (mm)				
	1.933	1.55				
	1.737	1.49				
	1.645	1.45				
	1.809	1.401				
	1.832	1.323				
	1.863	1.28				
	1.803	1.245				
	1.692	1.37				
	1.811	1.563				
	1.591	1.553				
**Average**	**1.772**	**1.423**				
**Standard Deviation**	**0.099**	**0.111**				
Maxillary (axial)	Line 1 to 2	Line 2 to 3				
	1.688	1.212				
	1.711	1.285				
	1.716	1.34				
	1.685	1.252				
	1.574	1.18				
	1.556	1.31				
	1.717	1.249				
	1.812	1.305				
	1.798	1.249				
	1.669	1.4				
**Average**	**1.693**	**1.278**				
**Standard Deviation**	**0.0818**	**0.0504**				
Dentary (coronal)	Region 1	Region 2	Region 2			

	Line 1 to 2	Line 1 to 2	Line 1 to 2			
	1.533	2.392	1.444			
	1.555	2.247	1.467			
	1.587	2.334	1.547			
	1.449	2.22	1.626			
	1.467	2.194	1.719			
	1.469	2.418	1.733			
	1.453	2.386	1.756			
	1.449	2.452	1.682			
	1.507	2.472	1.762			
	1.492	2.512	1.912			
**Average**	**1.496**	**2.363**	**1.665**			
**Standard Deviation**	**0.048**	**0.110**	**0.145**			
Dentary (axial)	Region 1		Region 2		Region 3	
	Line 1 to 2	Line 2 to 3	Line 1 to 2	Line 2 to 3	Line 1 to 2	Line 2 to 3
	2.085	1.821	1.612	2.166	1.89	1.486
	1.861	2.116	1.666	2.126	1.852	1.343
	1.901	2.254	1.673	2.023	2.013	1.38
	1.878	2.277	1.647	2.003	1.86	1.338
	1.958	2.116	1.609	1.996	1.798	1.345
	2.008	2.017	1.627	1.887	1.807	1.367
	2.12	2.015	1.64	1.913	1.791	1.467
	2.068	1.949	1.473	1.865	1.757	1.441
	2.175	1.982	1.628	1.815	1.762	1.447
	1.941	2.147	1.585	1.843	1.657	1.470
**Average**	**2.000**	**2.069**	**1.616**	**1.964**	**1.819**	**1.408**
**Standard Deviation**	**0.109**	**0.140**	**0.057**	**0.119**	**0.094**	**0.059**
**Total Maxillary Avg.**	**1.541**					
**Total Maxillary Std. Dev.**	**0.221**					

**Total Dentary Avg.**	**1.822**					
**Total Dentary Std. Dev.**	**0.307**					
**Total Average**	**1.736**					
**Total Standard Deviation**	**0.311**					

Supplemental figures and text

Tooth Sampling Protocol and Location:

The two teeth were sampled by Dr. You while visiting the Keck Paleoenvironmental and Environmental Stable Isotope Laboratory. He sampled the teeth my placing a piece of tape on the tooth enamel surface and making every millimetre the entire length of the tooth. These millimetre markings roughly corresponded to coloration bands and ridges that we interpret to represent perikymata. Care was made to avoid dentine during the sampling process. The tooth enamel is orange in color so as the drill started to approach the enamel-dentine interface, the color started to get very light. When this occurred, we stopped drilling to avoid sampling dentine. Sample lines can be found in Figure S1. The second half of the dentary tooth was processed incorrectly and thus, no samples were plotted from this region.

Tooth $\delta^{18}O_p$ outliers:

As is noted in the figures, three samples out of the 138 total number of samples are anomalously low relative to their sample neighbors (LZSM-3 LZSM-33, LZSD-59). These samples account for less than 2% of the total samples. Given that during the time of analysis, a bad electrical board on the IRMS at KPESIL occasionally caused spikes in the reference value (which for most samples, were tracked and those samples eliminated) it is likely that these three samples also represent instrumentation error. They were not initially removed from the data set because the reference CO O-isotope values were still within one standard deviation of the reference gas (for KPESIL, -3.50‰ V-SMOW). All samples that did have CO reference gas values that were outside the standard deviation of the reference values were eliminated. Even if these are not outliers, they represent short-term consumption of isotopically light water which may be due to a number of ecological reasons such as short-term melting events (snow-melt) or short-term migration.

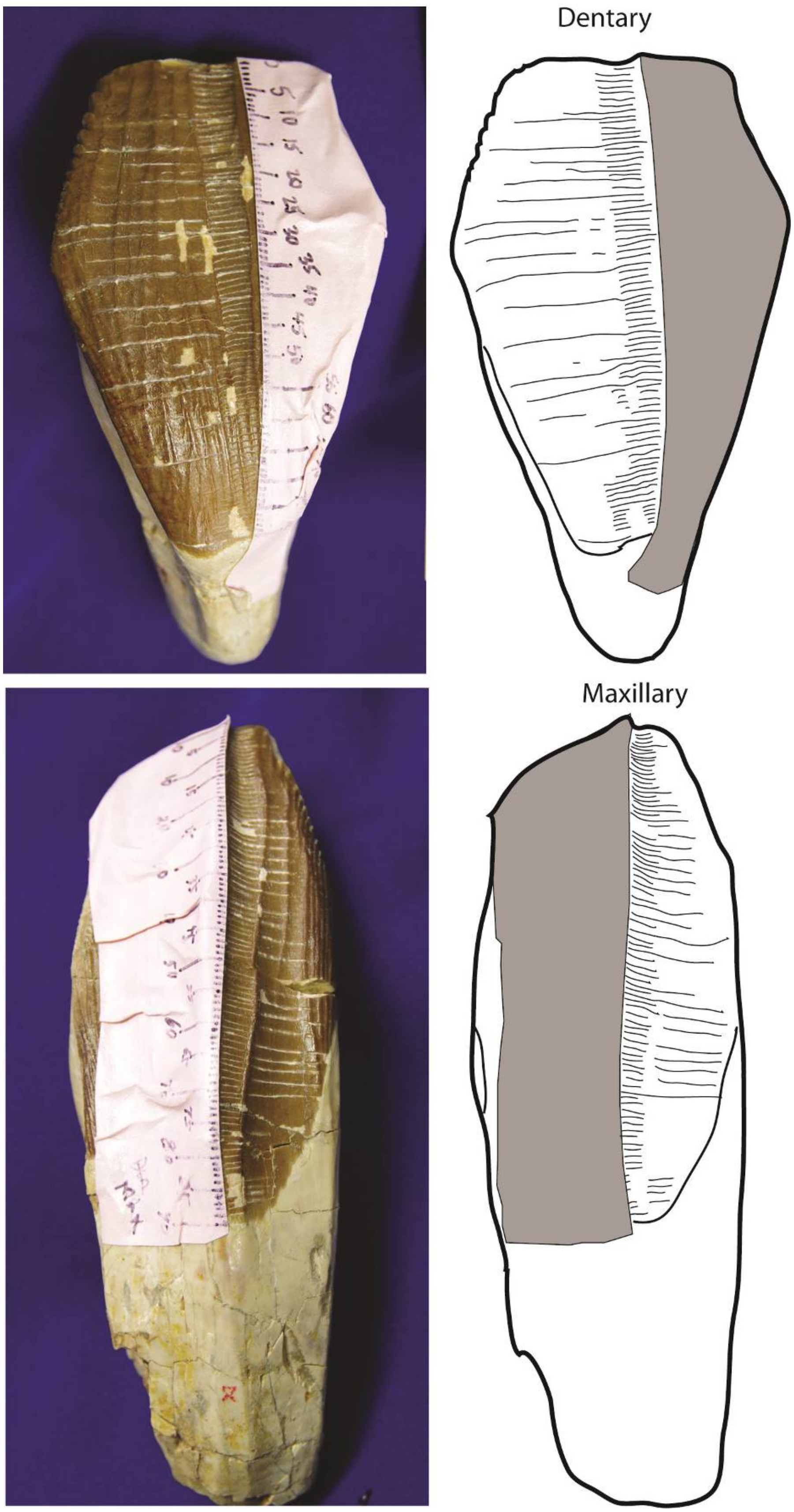

Figure S1. *Lanzhousaurus* teeth sampled in the study. Each drill line corresponds to 1 mm above the previous sample. Longer sample lines were generated to drill enough powder for carbonate isotope analysis.

Additional CT Images

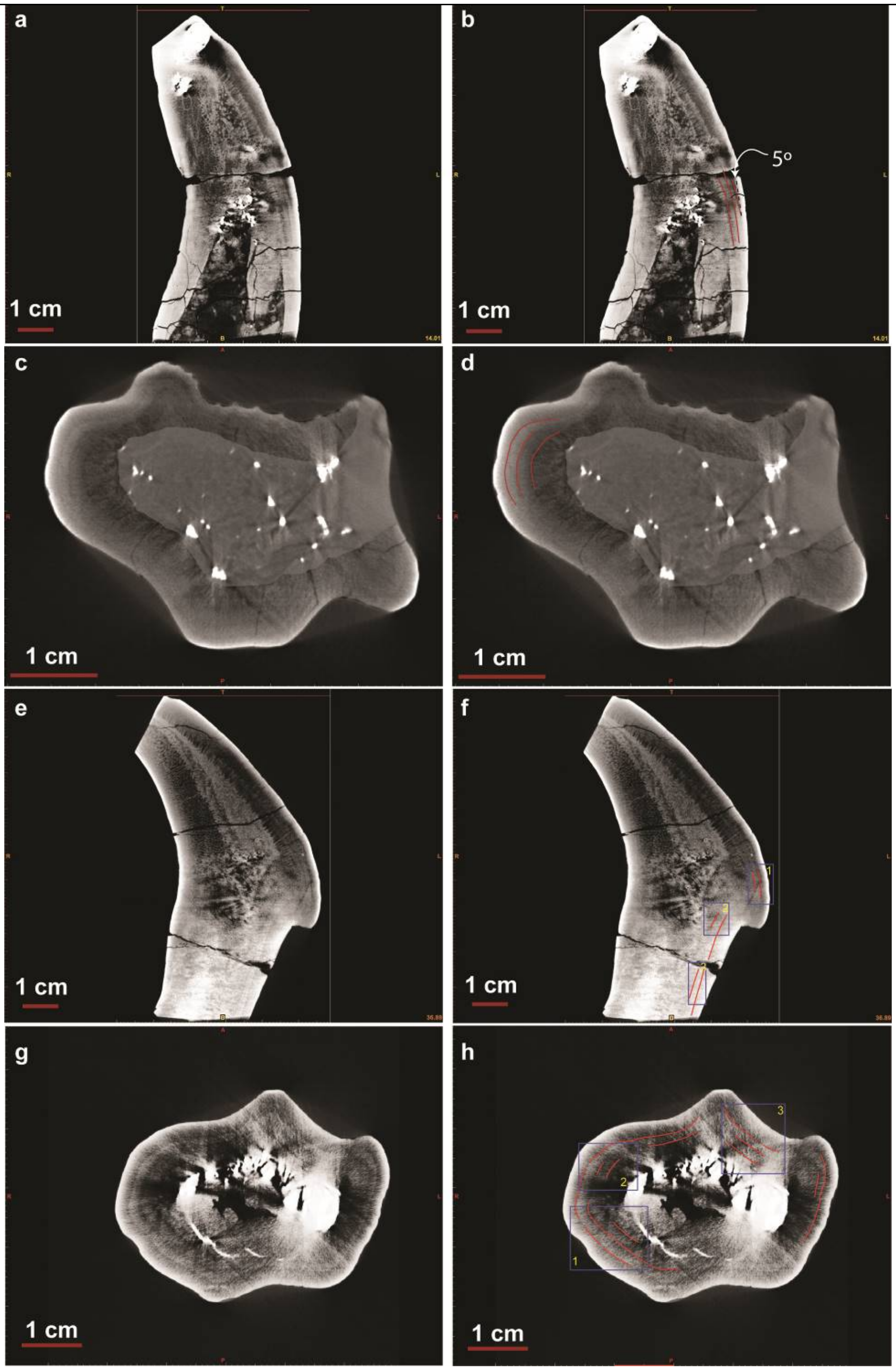

Figure S2. CT images of (a) maxillary tooth in coronal view, (b) interpreted incremental lines, (c) maxillary tooth in axial view, (d) interpreted incremental lines, (e) dentary tooth in coronal view, (f) interpreted incremental lines, (g) dentary tooth in axial view and (h) interpreted incremental lines. All lines were measured from the inner line (line 1) to the outer lines (lines 2 or 3 when three incremental lines could be viewed.

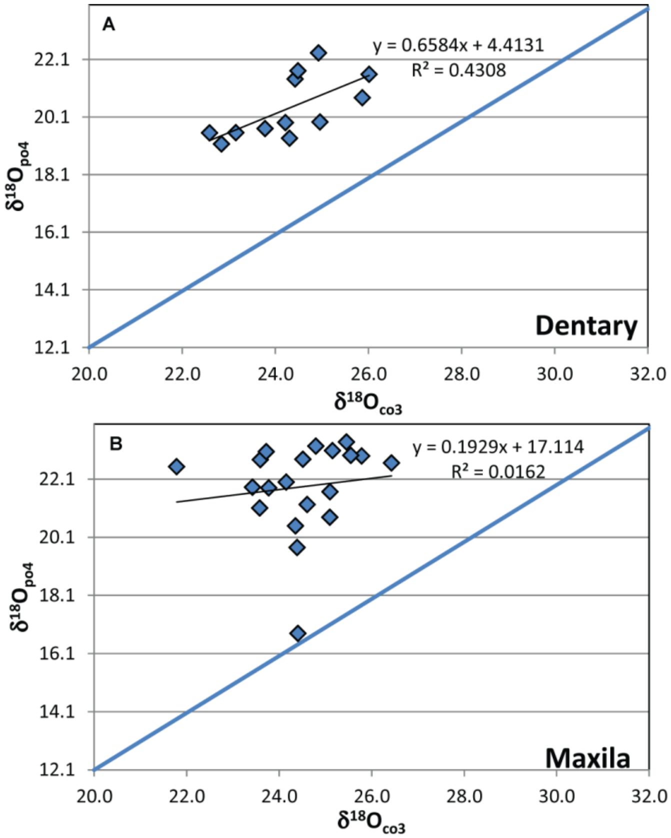

Figure S3. Stable isotopic composition of tooth enamel carbonate and phosphate relative to the Iacumin[1] equilibrium line for (**A**) the dentary tooth and (**B**) the maxillary tooth. Data plots off the line suggesting diagenesis to carbonate and or phosphate component of the enamel. Most likely, the carbonate oxygen component has undergone some degree of isotopic exchange with groundwater.

Supplemental Material References

1. Iacumin, P., Bocherens, H., Mariotti, A. & Longinelli, A. Oxygen isotope analyses of co-existing carbonate and phosphate in biogenic apatite: a way to monitor diagenetic alteration of bone phosphate? *Earth and Planetary Science Letters* **142,** 1–6 (1996).
